# How do immune cells shape type 1 diabetes? Insights from Mendelian randomization

**DOI:** 10.3389/fendo.2024.1402956

**Published:** 2024-12-24

**Authors:** Yunfeng Yu, Xinyu Yang, Juan Deng, Jingyi Wu, Siyang Bai, Rong Yu

**Affiliations:** ^1^ School of Traditional Chinese Medicine, Hunan University of Chinese Medicine, Changsha, Hunan, China; ^2^ Department of Endocrinology, The First Hospital of Hunan University of Chinese Medicine, Changsha, Hunan, China; ^3^ The Third School of Clinical Medicine, Zhejiang Chinese Medical University, Hangzhou, Zhejiang, China

**Keywords:** immune cell, phenotype, type 1 diabetes, GWAS, Mendelian randomization

## Abstract

**Objective:**

The role of immune cells in type 1 diabetes (T1D) is unclear. The aim of this study was to assess the causal effect of different immune cells on T1D using Mendelian randomization (MR).

**Methods:**

A dataset of immune cell phenotypes (numbered from GCST0001391 to GCST0002121) was obtained from the European Bioinformatics Institute, while a T1D dataset (numbered finngen_R10_T1D) was obtained from FinnGen. Single nucleotide polymorphisms meeting the conditions were screened stepwise according to the assumptions of association, independence, and exclusivity. Inverse variance weighted was used as the main method for the MR analysis. MR-Egger was used to assess the horizontal pleiotropy of the results. Cochran’s *Q* and the leave-one-out method were respectively used for the heterogeneity analysis and the sensitivity analysis of the results.

**Results:**

MR analysis showed that effector memory (EM) double-negative (DN) (CD4^−^CD8^−^) %T cells [odds ratio (OR) = 1.157, 95% confidence interval (95% CI) = 1.016–1.318, *p* = 0.028, false discovery rate (FDR) = 0.899], EM CD8^br^ %T cells (OR = 1.049, 95% CI = 1.003–1.098, *p* = 0.037, FDR = 0.902), CD28 on CD28^+^CD45RA^+^CD8^br^ (OR = 1.334, 95% CI = 1.132–1.571, *p* = 0.001, FDR = 0.044), IgD^+^CD38^dim^ %lymphocytes (OR = 1.045, 95% CI = 1.002–1.089, *p* = 0.039, FDR = 0.902), CD80 on monocytes (OR = 1.084, 95% CI = 1.013–1.161, *p* = 0.020, FDR = 0.834), SSC-A on plasmacytoid dendritic cells (pDCs) (OR = 1.174, 95% CI = 1.004–1.372, *p* = 0.044, FDR = 0.902), and FSC-A on pDCs (OR = 1.182, 95% CI = 1.011–1.382, *p* = 0.036, FDR = 0.902) were associated with an increased genetic susceptibility to T1D. Cochran’s *Q* showed that there was heterogeneity for CD28 on the CD28^+^CD45RA^+^CD8^br^ results (*p* = 0.043), whereas there was no heterogeneity for the other results (*p* ≥ 0.05). The sensitivity analysis showed that the MR analysis results were robust.

**Conclusion:**

The MR analysis demonstrated that seven immune cell phenotypes were associated with an increased genetic susceptibility to T1D. These findings provide a new direction for the pathogenesis of and the drug development for T1D.

## 1 Introduction

Diabetes is a chronic metabolic disease that has two main subtypes: type 1 diabetes (T1D) and type 2 diabetes (T2D) (1). T1D, also known as insulin-dependent diabetes, is caused by the destruction of β cells in the pancreas, resulting in an absolute lack of insulin, and is mainly seen in children and adolescents (2). It is generally characterized by the persistent elevation of blood glucose, polydipsia, polyphagia, polyuria, and weight loss (3). Although exogenous insulin supplementation controls the blood glucose levels and relieves the symptoms, it does not reverse or cure T1D (4). Moreover, as the disease progresses, many patients with T1D suffer from diabetic ketoacidosis, severe hypoglycemia, and cardiovascular complications (4). An epidemiological study indicated that T1D has become the third most common chronic disease in children, with more than 1.2 million children and adolescents worldwide suffering from T1D in 2021 (5). As the worldwide prevalence of T1D continues to rise, the direct and indirect healthcare costs it imposes on the country and society also continue to increase, and the resulting global public health problems are becoming increasingly serious (6).

Although the pathogenesis of T1D has not been fully elucidated, previous studies have suggested that genetic variants and environmental factors increase the genetic susceptibility to T1D by compromising immune homeostasis (7, 8). Previous research identified 26 genetic loci associated with the risk of T1D in genome-wide association studies (GWAS), 19 of which were associated with immune regulation (9, 10). Related studies have shown that, in addition to the crosstalk between β cells and the environment, a crosstalk between β cells and immunity also plays a key role in the development of T1D (8). For one, T lymphocytes are associated with the autoimmune damage to pancreatic β cells. A study conducted in the United States revealed that pancreatic β cells activate CD4^+^ T cells by releasing insulin peptide fragments into the bloodstream, subsequently triggering the specific recognition and attack of β cells by T cells (11). Another study from Argentina identified CD8^+^ T cells as a crucial contributor to T1D and a novel marker of β-cell autoimmunity, emphasizing the role of immune cells in T1D (12). For another, B lymphocytes also play a significant role in the autoimmune damage to pancreatic β cells. A study from the United Kingdom reported that, among the pancreatic lymphocytes of newly diagnosed T1D patients, B lymphocytes were present at a frequency second only to that of CD8^+^ T lymphocytes (13). This evidence underscores the intricate relationship between immune cells and T1D. However, the evidence to date is mainly observational and primarily concentrates on a few specific immune cells, which does not allow for a full elucidation of the causal relationship between different immune cells and T1D. Therefore, there is a need for novel and comprehensive approaches to assess the effects of different immune cell phenotypes on T1D.

Mendelian randomization (MR) is a novel approach to epidemiological studies that analyzes the causal effects of two factors through genetic variation. Since genotypes follow a random assignment principle in meiosis, MR is less susceptible to reverse causation and confounding variables than traditional methods (14, 15). This study assessed the impact of 731 immune cell phenotypes on the genetic susceptibility to T1D using GWAS data, aiming to identify key immune cells in the pathogenesis of T1D and to offer insights for its prevention and treatment.

## 2 Materials and methods

### 2.1 Study design

**Figure 1:**
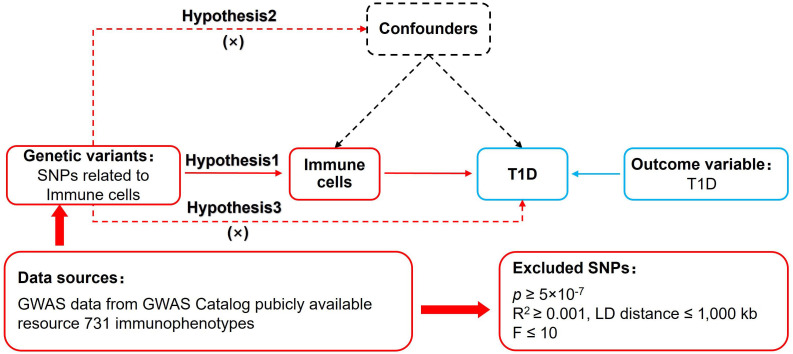
MR design for immune cells on genetic susceptibility to T1D. MR, Mendelian randomization; T1D, type 1 diabetes.

MR of immune cells and T1D was based on three fundamental assumptions (16) (**Figure 1**). The association assumption requires that single nucleotide polymorphisms (SNPs) are strongly correlated with exposure. The independence assumption requires that SNPs are independent of confounding variables. The exclusivity assumption requires that SNPs only act on outcomes through the exposure and not other pathways.

### 2.2 Data sources

The data sources for exposure and outcome are detailed in **Table 1**. Immune cell datasets numbered from GCST0001391 to GCST0002121 were acquired from the European Bioinformatics Institute (www.ebi.ac.uk/) (17). These datasets are from a study on 3,757 Sardinian residents that investigated the impact of approximately 22 million variants on 731 immune cell traits, which included 118 absolute cell counts, 389 mean fluorescence intensities of surface antigens, 32 morphological parameters, and 192 relative counts (17). The T1D dataset numbered finngen_R10_T1D was obtained from FinnGen (www.finngen.fi/fi), which contains genetic information on 339,432 Europeans. Due to the exposure data coming from the European Bioinformatics Institute and the outcome data coming from FinnGen, the overlap rate of the samples was extremely small. In addition, as the database is open access, no additional ethical review was required.

**Table 1:** Details of the genome-wide association studies (GWAS) included in the Mendelian randomization.

Year	Trait	GWAS ID	Population	Sample size	Web source
2023	Immune cells	GCST0001391 to GCST0002121	European	3,757	www.ebi.ac.uk/
2023	T1D	finngen_R10_T1D	European	339,432	www.finngen.fi/fi

*T1D*, type 1 diabetes.

### 2.3 Selection of genetic instrument variables

Firstly, due to the threshold of *p* < 5 × 10^−8^ being insufficient to obtain an adequate number of SNPs and exposures containing SNPs, we established a threshold of *p* < 5 × 10^−7^ to search for SNPs closely related to the exposure, thereby enhancing the statistical power and satisfying the association assumption (18, 19). Only 612 immune cells (87.32%) contained at least one SNP at the *p* < 5 × 10^−8^ threshold, while 726 immune cells (99.32%) contained at least one SNP at the *p* < 5 × 10^−7^ threshold. Secondly, restricted *R* ^2^ < 0.001 and *k*_b_ = 1,000 were used to search for independent SNPs to exclude interference from linkage disequilibrium. Thirdly, *F* > 10 was restricted to search for strongly correlated SNPs to exclude the interference of weakly correlated variables. *F* was calculated as *F* = [*R* ^2^/( $1-R^{2}$ )]*[ (N−K−1 )/ K ], where *K* is the number of paired samples, *N* is the total number of samples, and *R* ^2^ is the cumulative explained variance. Fourthly, SNPs containing confounding variables such as age, gender, body mass index, marital status, and education level were excluded using PhenoScanner and Google Scholar to fulfill the independence assumption. Fifthly, mismatched and duplicate SNPs were excluded based on the effect allele frequency when adjusting the allele orientation for exposure and outcome. Lastly, the MR–pleiotropy residual sum and outlier (MR-PRESSO) method was used to exclude SNPs with significant bias (*p* < 1) to ensure the correctness of causal inference.

### 2.4 Data analysis

STROBE-MR was used as a guiding methodology (20). R 4.3.1 with the TwoSampleMR (0.5.7) program package installed was used to perform the operations for MR analysis. Firstly, inverse variance weighted (IVW) was set as the main evaluation tool as it allows for an unbiased causal analysis without pleiotropy. Weighted median, which is sensitive to outliers, and MR-Egger, which analyzes data in the presence of pleiotropy, were set as the secondary assessment tools. The statistical significance was set to *p* < 0.05. In addition, MR-Egger was used to analyze horizontal pleiotropy, which was required to satisfy the exclusivity assumption (*p* ≥ 0.05). Secondly, false discovery rate (FDR) based on the Benjamini–Hochberg method was used to correct the MR analysis results, with *p* < 0.05 considered a significant correlation and *p* ≥ 0.05 considered a potential correlation. Thirdly, Cochran’s *Q* and the leave-one-out method were used to analyze heterogeneity and sensitivity, respectively. There was no heterogeneity in the results when *p* ≥ 0.05, and the results were robust when no significant changes in the combined effect sizes were observed.

## 3 Results

### 3.1 Genetic instrument variables

We screened SNPs that met the basic assumptions of MR according to the steps enumerated above and excluded SNPs related to the confounding variables. Among them, effector memory (EM) CD8^br^ %T cells excluded rs191753228 (cigarette consumption), rs76668354 (biological sex), and rs4507432 (body mass index); IgD^+^CD38^dim^ %lymphocytes excluded rs16974449 (body mass index); CD80 on monocytes excluded rs114253672 (smoking initiation) and rs35075155 (body mass index); and SSC-A on plasmacytoid dendritic cells (pDCs) excluded rs17552904 (smoking initiation). The included exposures and SNPs are shown in **Supplementary Table S1**.

### 3.2 Two-sample MR analysis

**Figure 2:**
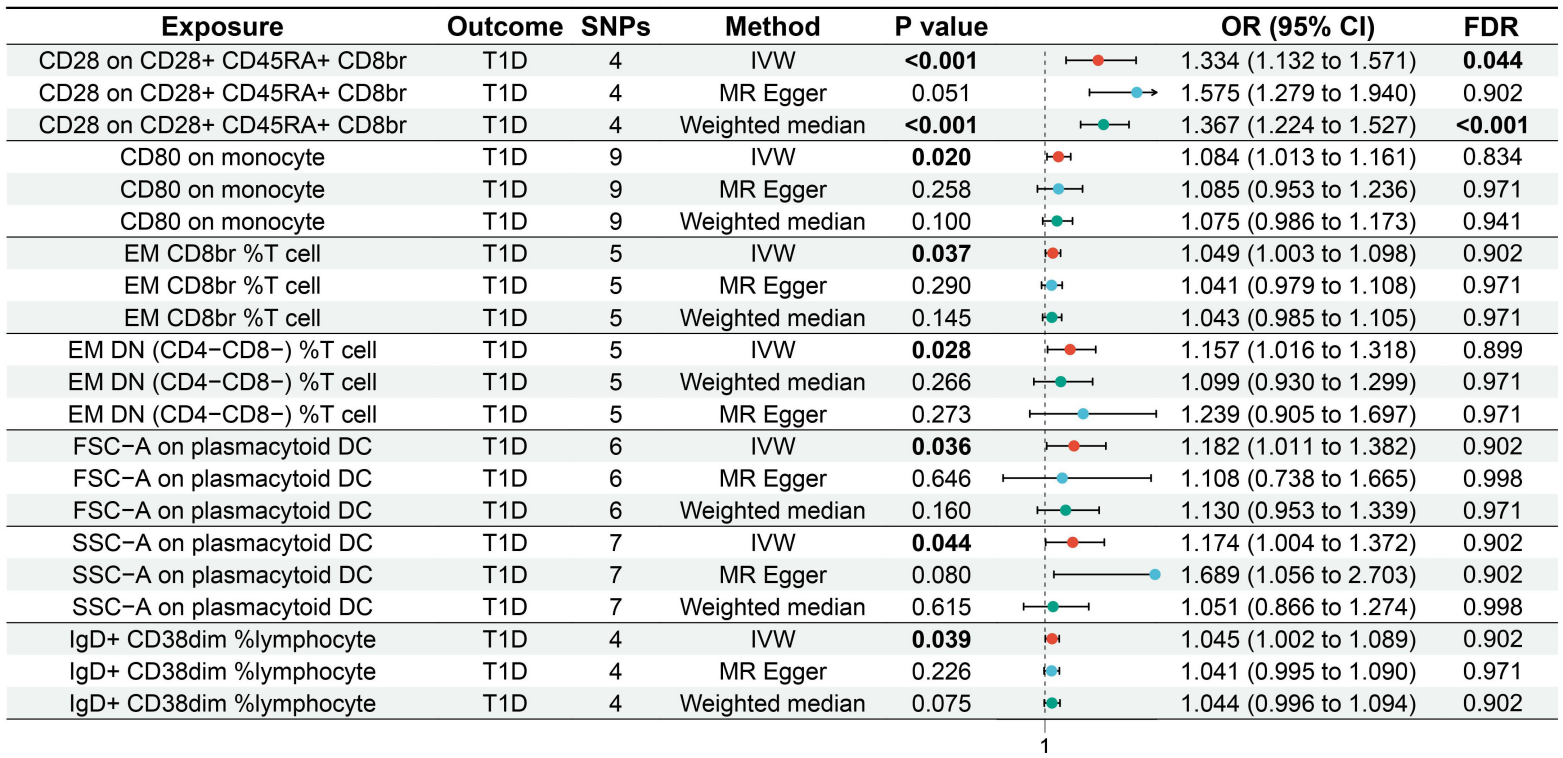
Forest plot of the MR analysis for immune cells on genetic susceptibility to T1D. MR, Mendelian randomization; T1D, type 1 diabetes.

**Figure 3:**
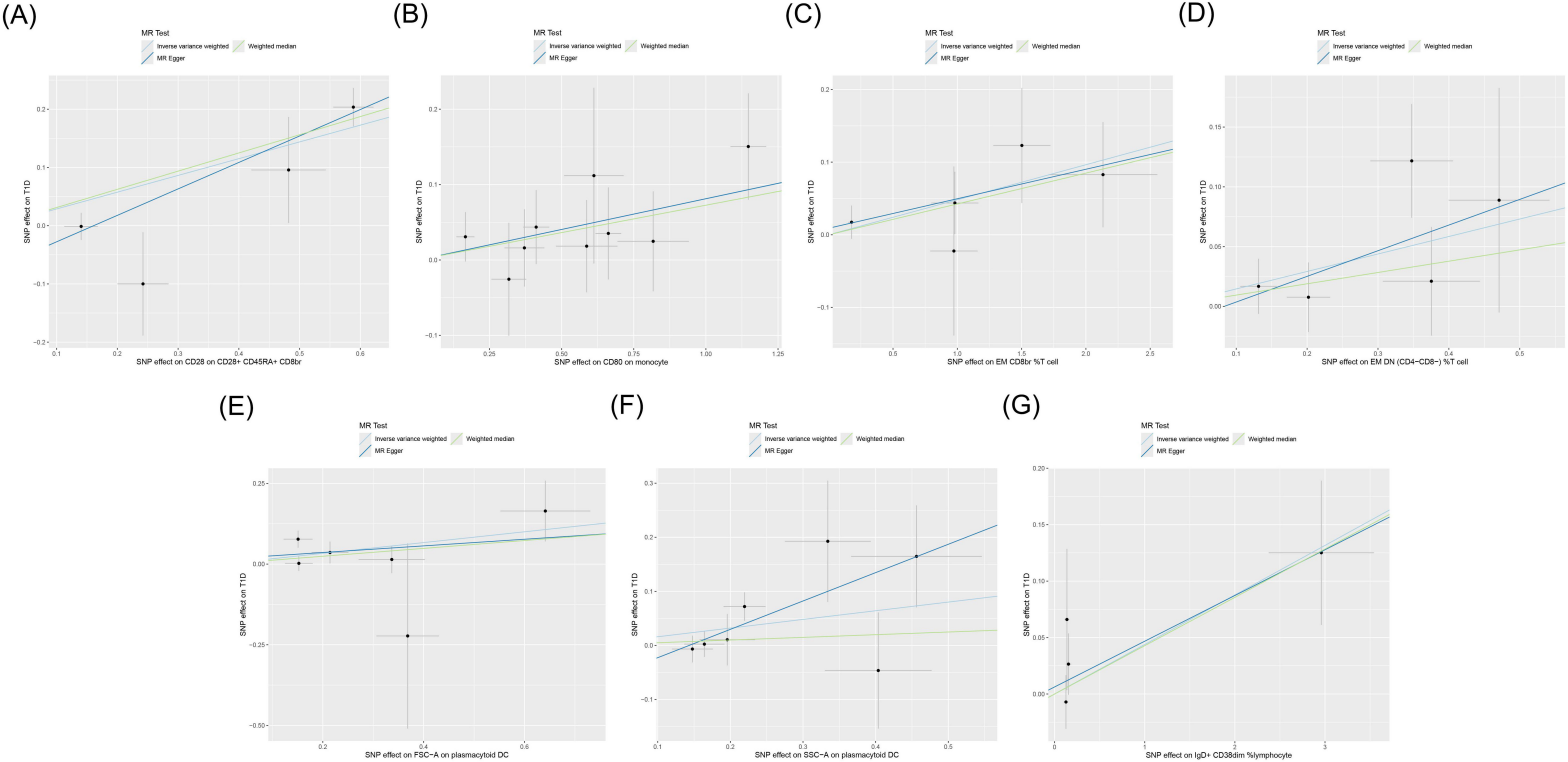
Scatter plot of the MR analysis for immune cells on genetic susceptibility to T1D. MR, Mendelian randomization; T1D, type 1 diabetes. **(A)** CD28 on CD28^+^CD45RA^+^CD8^br^ in T1D. **(B)** CD80 on monocytes in T1D. **(C)** Effector memory (EM) CD8^br^ %T cells in T1D. **(D)** EM double-negative (DN) (CD4^−^CD8^−^) %T cells in T1D. **(E)** FSC-A on plasmacytoid dendritic cells (pDCs) in T1D. **(F)** SSC-A on pDCs in T1D. **(G)** IgD+ CD38^dim^ %lymphocytes in T1D. MR, Mendelian randomization; T1D, type 1 diabetes.

IVW showed that EM double-negative (DN) (CD4^−^CD8^−^) %T cells [odds ratio (OR) = 1.157, 95% confidence interval (95% CI) = 1.016–1.318, *p* = 0.028, FDR = 0.899], EM CD8^br^ %T cells (OR = 1.049, 95% CI = 1.003–1.098, *p* = 0.037, FDR = 0.902), CD28 on CD28^+^CD45RA^+^CD8^br^ (OR = 1.334, 95% CI = 1.132–1.571, *p* < 0.001, FDR = 0.044), IgD^+^CD38^dim^ %lymphocytes (OR = 1.045, 95% CI = 1.002–1.089, *p* = 0.039, FDR = 0.902), CD80 on monocytes (OR = 1.084, 95% CI = 1.013–1.161, *p* = 0.020, FDR = 0.834), SSC-A on pDCs (OR = 1.174, 95% CI = 1.004–1.372, *p* = 0.044, FDR = 0.902), and FSC-A on pDCs (OR = 1.182, 95% CI = 1.011–1.382, *p* = 0.036, FDR = 0.902) were associated with an increased genetic susceptibility to T1D. The forest plot and the scatter plot are shown in **Figures 2** and **3**, respectively. MR-Egger showed no horizontal pleiotropy for these results (*p* ≥ 0.05), as shown in **Supplementary Table S2**.

### 3.3 FDR analysis

FDR analysis showed that CD28 on CD28^+^CD45RA^+^CD8^br^ was significantly associated with genetic susceptibility to T1D (*p* = 0.044), while EM DN (CD4^−^CD8^−^) %T cells (*p* = 0.899), EM CD8^br^ %T cells (*p* = 0.902), IgD^+^CD38^dim^ %lymphocytes (*p* = 0.902), CD80 on monocytes (*p* = 0.834), SSC-A on pDCs (*p* = 0.902), and FSC-A on pDCs (*p* = 0.902) were potentially associated with genetic susceptibility to T1D.

### 3.4 Heterogeneity and sensitivity analyses

**Figure 4:**
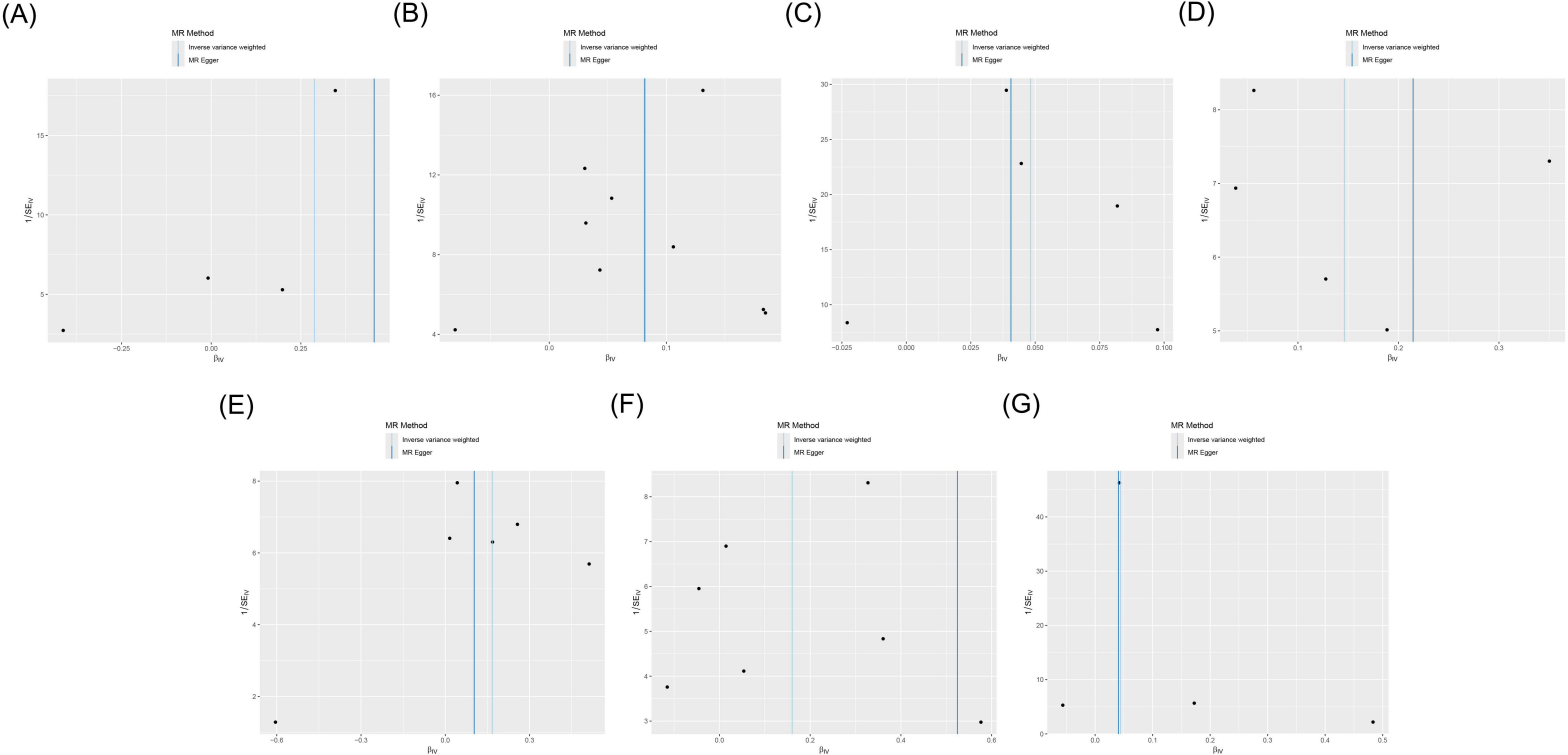
Funnel plot of the MR analysis for immune cells on genetic susceptibility to T1D. **(A)** CD28 on CD28^+^CD45RA^+^CD8^br^ in T1D. **(B)** CD80 on monocytes in T1D. **(C)** Effector memory (EM) CD8^br^ %T cells in T1D. **(D)** EM double-negative (DN) (CD4^−^CD8^−^) %T cells in T1D. **(E)** FSC-A on plasmacytoid dendritic cells (pDCs) in T1D. **(F)** SSC-A on pDCs in T1D. **(G)** IgD+ CD38^dim^ %lymphocytes in T1D. MR, Mendelian randomization; T1D, type 1 diabetes.

**Figure 5:**
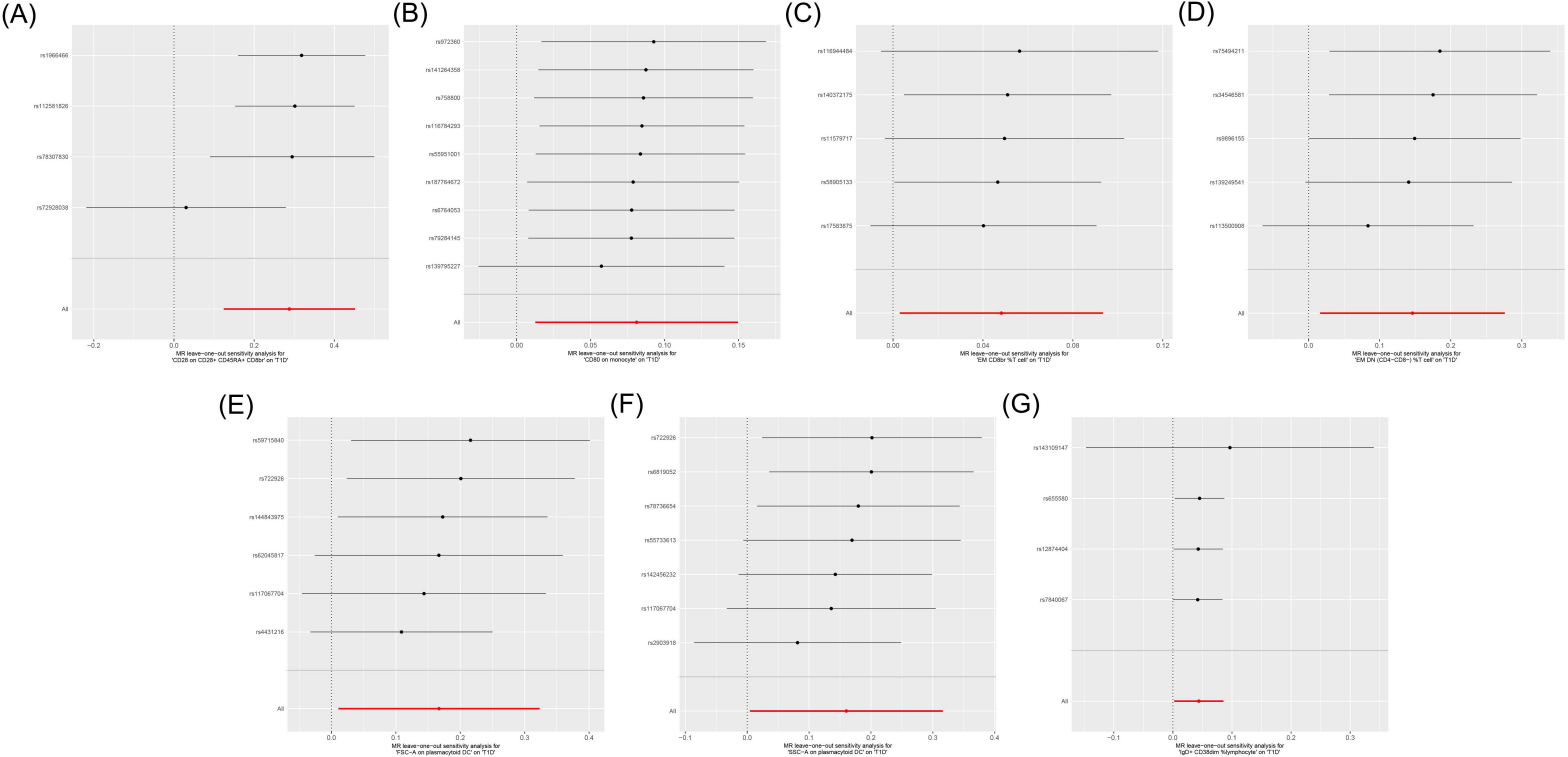
Leave-one-out sensitive analysis for immune cells on genetic susceptibility to T1D. **(A)** CD28 on CD28^+^CD45RA^+^CD8^br^ in T1D. **(B)** CD80 on monocytes in T1D. **(C)** Effector memory (EM) CD8^br^ %T cells in T1D. **(D)** EM double-negative (DN) (CD4^−^CD8^−^) %T cells in T1D. **(E)** FSC-A on plasmacytoid dendritic cells (pDCs) in T1D. **(F)** SSC-A on pDCs in T1D. **(G)** IgD+ CD38^dim^ %lymphocytes in T1D. T1D, type 1 diabetes.

Cochran’s *Q* test showed heterogeneity in the results of CD28 on CD28^+^CD45RA^+^CD8^br^ (*p* = 0.043), whereas no significant heterogeneity was observed in the other results (*p* ≥ 0.05), as shown in **Supplementary Table S3** and **Figure 4**. Sensitivity analysis demonstrated that the MR analysis results were robust, as illustrated in **Figure 5**.

## 4 Discussion

Immune cells reside in tissues and organs within the body, the purpose of which is to perform immune functions and regulate immune homeostasis. Similar to the immune cells in other tissues, those residing in pancreatic islets play a crucial role in the maintenance of immune homeostasis within the islet microenvironment. A previous study has identified differences in the immune cell phenotypes between patients with T1D and the general population (21). However, limited both in quantity and quality of evidence, it is currently challenging to ascertain the roles of various immune cells in the onset process of T1D. To our knowledge, this study represents the first comprehensive assessment of the causal effects of 731 immune cells on T1D. The results revealed that CD28 on CD28^+^CD45RA^+^CD8^br^ was significantly associated with genetic susceptibility to T1D, while EM DN (CD4^−^CD8^−^) %T cells, EM CD8^br^ %T cells, IgD^+^CD38^dim^ %lymphocytes, CD80 on monocytes, SSC-A on pDCs, and FSC-A on pDCs were potentially associated with genetic susceptibility to T1D. Cochran’s *Q* test showed heterogeneity in the results of CD28 on CD28^+^CD45RA^+^CD8^br^, which may be related to the sources of the exposure and outcome. In this MR analysis, the dataset of exposure was from Italians and the dataset of outcome from Finns. Although both Italians and Finns are Europeans, long-term geographical isolation may have resulted in genetic differences between the Finns living in Northern Europe and the Italians living in Southern Europe. However, despite the fact that there was heterogeneity in the results of CD28 on CD28^+^CD45RA^+^CD8^br^, the sensitivity analysis suggested the robustness of these findings.

EM DN (CD4^−^CD8^−^) %T cells comprise the proportion of EM T lymphocytes with no expression of CD4 and CD8 out of the total T lymphocytes. DN T cells exhibit distinct effector functions compared with CD4 T cells, amplifying interleukin 2 (IL-2) production (22). Furthermore, overactivated IL-2 might exacerbate the autoimmune response against pancreatic β cells (23). In addition, DN T cells generally refer to CD3^+^ T lymphocytes with a memory function. CD3 is a cell surface marker molecule that plays a key role in the differentiation, development, and maturation of T cells. During T-cell development, CD3 molecules bind to T-cell receptors (TCRs) to form the TCR/CD3 complex, which, together, participate in signaling and activation. A study has shown that oral administration of anti-CD3 drugs delayed the onset of diabetes and reduced morbidity in non-obese diabetic (NOD) mice, the mechanism of which may be related to the regulation of interferon gamma (IFN-γ) and IL-17 (24). Since IFN-γ and IL-17-mediated pro-inflammatory pathways play a key role in β-cell deletion, blockage of the secretion of IFN-γ and IL-17 helps to delay or reverse the pathogenesis of T1D (25). These pieces of evidence support the association of DN T cells with the increased risk of diabetes, pointing to EM DN (CD4^−^CD8^−^) %T cells as a potential risk factor for T1D.

EM CD8^br^ %T cells represent the proportion of EM T lymphocytes with a high CD8 expression in T lymphocytes. CD8^+^ T cells comprise the main driving factor for the destruction of pancreatic β cells. They specifically recognize and attack pancreatic β cells, thereby leading to insufficient insulin secretion (26). In addition, CD8^+^ T cells secrete cytokines such as perforin, tumor necrosis factor (TNF), IFN-γ, and IL-2 (27), and the direct action of these cytokines is a key factor leading to β-cell death (28, 29). Relevant studies have shown that pancreatic β-cell death is inhibited by the depletion of CD8 T cells, thereby ameliorating the condition of T1D (30). Previous studies have confirmed the significant infiltration of CD8^+^ T cells in the pancreas of patients with T1D (31), with the percentage and the absolute count of late peripheral blood EM CD8^+^ T cells being markedly elevated compared with those in healthy individuals (32). These pieces of evidence suggest EM CD8^br^ %T cells as a potential risk factor for T1D.

CD28 on CD28^+^CD45RA^+^CD8^br^ comprise a cell subset characterized by the expression of CD28, CD45RA, and a high level of CD8. CD28 belongs to the immunoglobulin superfamily, and nearly half of them are expressed on CD8^+^ T cells, serving as co-stimulatory molecules essential for the maintenance of T-cell activation. By binding to B7-1 (CD80) and B7-2 (CD86) on antigen-presenting cells, CD28 provides co-stimulatory signals for optimal T-cell activation (33). Related studies have shown that blockage of CD28 co-stimulation can limit T-cell activation and infiltration in pancreatic islets, thereby preventing and treating T1D (34). CD45RA is an elongated isoform of CD45 that is typically expressed on the surface of naive T cells. After antigen stimulation, CD8^+^CD45RA^+^ T cells will transform into TEMRA cells (EM cells that re-express CD45RA) with characteristics of memory T cells and effector T cells (35). TEMRA cells are cytotoxic and can secrete cytokines such as perforin to induce the apoptosis of pancreatic β cells (36). A clinical study conducted in Spain confirmed the connection between TEMRA cells and T1D, reporting that the peripheral blood TEMRA levels in patients with T1D were significantly higher than those in healthy individuals (32). These findings suggest the pivotal roles of CD28, CD45RA, and CD8 in the progression of T1D, supporting CD28 on CD28^+^CD45RA^+^CD8^br^ as a potential risk factor for T1D.

IgD^+^CD38^dim^ %lymphocytes comprise the proportion of lymphocytes with immunoglobulin D (IgD) present on the surface and with a low CD38 expression out of the total lymphocytes. IgD is an antibody in the human immune system that primarily exists in membrane-bound form on the surface of B cells, playing a role in the activation and maturation of B cells. In pancreatic lymphocytes from newly diagnosed T1D patients, B lymphocytes are present at a frequency second only to CD8T lymphocytes (13). After treatment with rituximab, the destruction of pancreatic β cells decreases with the selective depletion of B lymphocytes, thereby preserving the function of pancreatic β cells in newly diagnosed T1D patients as much as possible (37). In addition to B lymphocytes, CD38 also plays a significant role in T1D. A study of Finnish children demonstrated that the anti-CD38 antibody levels increased with disease duration 10 years after the diagnosis of T1D (38). CD38 serves as the primary NAD-consuming enzyme, maintaining cellular NAD^+^ homeostasis. NAD, a crucial cofactor in the energy metabolism regulation, is involved in processes such as glycolysis, β-oxidation, and oxidative phosphorylation (39). In chronic T1D animal models such as streptozotocin diabetic rats, the NAD levels exhibited a significant decline (40). A related study indicated that high-dose nicotinamide prevents or delays T1D by modulating CD38 and reducing NAD^+^ depletion to protect pancreatic β cells (41). These pieces of evidence suggest that IgD and CD38 contribute to the progression of T1D, indirectly supporting IgD^+^CD38^dim^ %lymphocytes as a potential risk factor for T1D.

In addition to lymphocytes, the role of myeloid antigen-presenting cells, such as monocytes, macrophages, and DCs, in T1D has been well documented (42). Monocytes are precursor cells produced by the bone marrow that enter tissues through the circulation and differentiate to form macrophages and DCs (43). In this MR analysis, it was identified that the myeloid cell immunophenotypes CD80 on monocytes, human leukocyte antigen DR isotype (HLA-DR) on CD33^br^HLA DR^+^CD14^−^, SSC-A on pDCs, and FSC-A on pDCs were associated with an increased risk of T1D. CD80 on monocytes refer to monocytes with surface expression of CD80. CD80, also known as B7-1, is widely expressed on the surface of a variety of immune cells and plays a crucial regulatory role in immune response. The expression of CD80 synergizes with pancreas-specific cytotoxic T cells in the rapid destruction of β cells (44). When CD80 was co-expressed with TNF-α or IL-2, a large proportion of mice developed severe pancreatitis and diabetes mellitus (45, 46). In addition, when CD80 was expressed on pancreatic β cells from B6 mice backcrossed once with genetically susceptible NOD, the onset rate of diabetes and the severity of the autoimmune response also increased (47). This suggests that CD80 on monocytes may contribute to the pathogenesis of T1D. HLA-DR on CD33^br^HLA DR^+^CD14^−^ represents a subpopulation of immune cells with positive HLA-DR, high expression of CD33, and no expression of CD14. Specific HLA-DR genotypes are associated with an increased genetic susceptibility to T1D, as mentioned above. CD33 is a cell surface molecule expressed widely in bone marrow hematopoietic cells that is involved in the regulation of inflammation, activation of cell proliferation, and immune response (48, 49). The current study demonstrated that CD33 HLA-DR myeloid-derived suppressor cells (MDSCs) were significantly increased in the peripheral blood of patients with T1D and positively correlated with the levels of HbA1C (50). These pieces of evidence support that CD80 on monocytes and HLA-DR on CD33^br^HLA DR^+^CD14^−^ could serve as risk factors for T1D.

SSC-A on pDCs and FSC-A on pDCs are two phenotypes of DCs. SSC-A is a parameter used in flow cytometry to characterize the side scatter properties of a cell, which is commonly used to assess the intracellular complexity and particle content. FSC-A is a parameter used in flow cytometry to characterize the forward scatter signal of cells, typically employed to assess the size and morphology of cells. SSC-A on pDCs and FSC-A on pDCs may refer to pDCs with specific scatter properties that act as a bridge between innate and adaptive immunity in T1D development (51). pDCs are predominantly found in the peripheral blood, lymph, and bone marrow, playing roles in the secretion of IFN, the modulation of T-cell immune responses, and in autoimmune diseases (51). A clinical study in the United States showed that the number of pDCs was significantly increased in the peripheral blood of patients with T1D compared with healthy subjects, while that of other DC types remained similar (51). Furthermore, in the NOD mouse model, the injection of IFN-α or its inducers significantly accelerated the onset of T1D (52), while the depletion of pDCs significantly delayed the onset of T1D (53). These pieces of evidence support that pDCs are strongly associated with T1D, indicating that SSC-A on pDCs and FSC-A on pDCs are potential risk factors for T1D.

While this MR analysis provides genetic insights into the causal relationship between immune cell phenotypes and T1D, certain limitations must be acknowledged. Firstly, the socioeconomic status of diverse racial groups significantly contributes to health disparities (54). The GWAS data included in this study were obtained from European populations in high-income countries, restricting the generalization of the findings to low-/middle-income countries and other ethnicities. Secondly, the heterogeneity of the results of naive CD8^br^ %T cells and HLA-DR on CD33^br^HLA DR^+^CD14^−^ might amplify the potential risk of bias. Thirdly, although this study identified the causal effects of eight immune cell phenotypes on T1D, the biological mechanisms through which they influence T1D remain unclear. Consequently, we anticipate the following improvements in future studies. Firstly, we should continuously enrich the GWAS data and make efforts to realize MR analysis for diverse ethnic and socioeconomic status groups. Secondly, we look forward to validate the expression levels of the immune cells identified in this study through animal experiments in order to provide more robust biological evidence. Thirdly, the design of high-quality clinical research protocols is expected to enrich the evidence-based support for the causal effect of immune cell phenotypes on T1D.

## 5 Conclusion

The MR analysis revealed that CD28 on CD28^+^CD45RA^+^CD8^br^ was significantly associated with genetic susceptibility to T1D, while EM DN (CD4^−^CD8^−^) %T cells, EM CD8^br^ %T cells, IgD^+^CD38^dim^ %lymphocytes, CD80 on monocytes, SSC-A on pDCs, and FSC-A on pDCs were potentially associated with genetic susceptibility to T1D. These findings provide a new direction for the pathogenesis of and the development of new drugs for T1D. However, further biological studies are necessary in the future to reveal their relationships and mechanisms.

## Data Availability

The original contributions presented in the study are included in the article/**Supplementary Material**. Further inquiries can be directed to the corresponding author.
