# Supplementary material for: How do immune cells shape type 1 diabetes? Insights from Mendelian randomization
Source: Front Endocrinol (Lausanne). 2024 Dec 24;15:1402956. doi: 10.3389/fendo.2024.1402956 (PMC11703746; doi:10.3389/fendo.2024.1402956)
Supplement: Supplementary file 1 [file Table1.docx]

**Supplementary Table S1.1** Information of identified SNPs in exposure (CD28 on CD28+ CD45RA+ CD8br) and outcomes (T1D).

|  |  | | | **Exposure (CD28 on CD28+ CD45RA+ CD8br)** | | |  | **Outcome (T1D)** | | | | |
| --- | --- | --- | --- | --- | --- | --- | --- | --- | --- | --- | --- | --- |
|  | **SNP** | **EA** | **OA** | **β** | **SE** | ***p* value** |  | **Case** | **Control** | **β** | **SE** | ***p* value** |
| 1 | rs112581826 | A | G | -0.2422 | 0.04219 | 9.43E-09 |  | 4,320 | 335,112 | 0.0999458 | 0.0887024 | 0.259847 |
| 2 | rs1966466 | A | G | 0.1404 | 0.02771 | 4.05E-07 |  | 4,320 | 335,112 | -0.00122755 | 0.0233058 | 0.957994 |
| 3 | rs72928038 | A | G | 0.5885 | 0.03315 | 1.64E-70 |  | 4,320 | 335,112 | 0.203638 | 0.0330253 | 7.00E-10 |
| 4 | rs78307830 | C | G | 0.4819 | 0.06136 | 4.04E-15 |  | 4,320 | 335,112 | 0.0957247 | 0.0910665 | 0.293189 |

SNP, single nucleotide polymorphism; EA, effect allele; OA, other allele; SE, standard error; T1D, type 1 diabetes.

**Supplementary Table S1.2** Information of identified SNPs in exposure (CD80 on monocyte) and outcomes (T1D).

|  |  | | | **Exposure (CD80 on monocyte)** | | |  | **Outcome (T1D)** | | | | |
| --- | --- | --- | --- | --- | --- | --- | --- | --- | --- | --- | --- | --- |
|  | **SNP** | **EA** | **OA** | **β** | **SE** | ***p* value** |  | **Case** | **Control** | **β** | **SE** | ***p* value** |
| 1 | rs116784293 | C | G | -0.3172 | 0.06051 | 1.59E-07 |  | 4,320 | 335,112 | 0.025499 | 0.0749893 | 0.733829 |
| 2 | rs139795227 | C | A | -1.147 | 0.06195 | 1.56E-76 |  | 4,320 | 335,112 | -0.150473 | 0.0706288 | 0.0331322 |
| 3 | rs141264358 | C | T | -0.5868 | 0.1067 | 3.81E-08 |  | 4,320 | 335,112 | -0.0183279 | 0.0612092 | 0.764612 |
| 4 | rs187764672 | T | C | -0.4118 | 0.04505 | 6.19E-20 |  | 4,320 | 335,112 | -0.0436292 | 0.0490749 | 0.373986 |
| 5 | rs55951001 | T | C | -0.371 | 0.06911 | 7.95E-08 |  | 4,320 | 335,112 | -0.0160391 | 0.0513173 | 0.754625 |
| 6 | rs6764053 | T | C | -0.1666 | 0.03139 | 1.11E-07 |  | 4,320 | 335,112 | -0.0307559 | 0.0328148 | 0.348626 |
| 7 | rs758800 | C | T | 0.6619 | 0.04462 | 8.81E-50 |  | 4,320 | 335,112 | 0.0352572 | 0.061113 | 0.563995 |
| 8 | rs79284145 | A | G | -0.6119 | 0.1031 | 2.94E-09 |  | 4,320 | 335,112 | -0.111872 | 0.116595 | 0.33731 |
| 9 | rs972360 | T | G | -0.8173 | 0.124 | 4.36E-11 |  | 4,320 | 335,112 | -0.0247794 | 0.0662933 | 0.708564 |

SNP, single nucleotide polymorphism; EA, effect allele; OA, other allele; SE, standard error; T1D, type 1 diabetes.

**Supplementary Table S1.3** Information of identified SNPs in exposure (EM CD8br %T cell) and outcomes (T1D).

|  |  | | | **Exposure (EM CD8br %T cell)** | | |  | **Outcome (T1D)** | | | | |
| --- | --- | --- | --- | --- | --- | --- | --- | --- | --- | --- | --- | --- |
|  | **SNP** | **EA** | **OA** | **β** | **SE** | ***p* value** |  | **Case** | **Control** | **β** | **SE** | ***p* value** |
| 1 | rs11579717 | A | G | -0.981 | 0.1833 | 8.70E-08 |  | 4,320 | 335,112 | -0.0437433 | 0.0429961 | 0.308973 |
| 2 | rs116944484 | A | G | -2.133 | 0.4227 | 4.51E-07 |  | 4,320 | 335,112 | -0.0827585 | 0.0724051 | 0.253042 |
| 3 | rs140372175 | C | A | -0.9734 | 0.1846 | 1.34E-07 |  | 4,320 | 335,112 | 0.0223265 | 0.11633 | 0.847802 |
| 4 | rs17583875 | A | G | -1.502 | 0.2221 | 1.35E-11 |  | 4,320 | 335,112 | -0.122939 | 0.0792173 | 0.120681 |
| 5 | rs58905133 | G | A | 0.1775 | 0.02609 | 1.02E-11 |  | 4,320 | 335,112 | 0.0172996 | 0.0229532 | 0.451036 |

SNP, single nucleotide polymorphism; EA, effect allele; OA, other allele; SE, standard error; T1D, type 1 diabetes.

**Supplementary Table S1.4** Information of identified SNPs in exposure (EM DN (CD4-CD8-) %T cell) and outcomes (T1D).

|  |  | | | **Exposure (EM DN (CD4-CD8-) %T cell)** | | |  | **Outcome (T1D)** | | | | |
| --- | --- | --- | --- | --- | --- | --- | --- | --- | --- | --- | --- | --- |
|  | **SNP** | **EA** | **OA** | **β** | **SE** | ***p* value** |  | **Case** | **Control** | **β** | **SE** | ***p* value** |
| 1 | rs113500908 | T | C | 0.3476 | 0.05835 | 2.57E-09 |  | 4,320 | 335,112 | 0.121719 | 0.0475955 | 0.0105465 |
| 2 | rs139249541 | G | A | 0.4709 | 0.07117 | 3.68E-11 |  | 4,320 | 335,112 | 0.0888334 | 0.0939203 | 0.344231 |
| 3 | rs34546581 | A | G | 0.2019 | 0.03063 | 4.35E-11 |  | 4,320 | 335,112 | 0.00768936 | 0.0291062 | 0.791639 |
| 4 | rs75494211 | A | G | 0.3753 | 0.06853 | 4.34E-08 |  | 4,320 | 335,112 | 0.0210672 | 0.0454203 | 0.642771 |
| 5 | rs9896155 | G | A | 0.1315 | 0.02587 | 3.71E-07 |  | 4,320 | 335,112 | 0.0167936 | 0.0230564 | 0.466388 |

SNP, single nucleotide polymorphism; EA, effect allele; OA, other allele; SE, standard error; T1D, type 1 diabetes.

**Supplementary Table S1.5** Information of identified SNPs in exposure (FSC-A on plasmacytoid DC) and outcomes (T1D).

|  |  | | | **Exposure (FSC-A on plasmacytoid DC)** | | |  | **Outcome (T1D)** | | | | |
| --- | --- | --- | --- | --- | --- | --- | --- | --- | --- | --- | --- | --- |
|  | **SNP** | **EA** | **OA** | **β** | **SE** | ***p* value** |  | **Case** | **Control** | **β** | **SE** | ***p* value** |
| 1 | rs117067704 | G | A | 0.6413 | 0.08914 | 6.28E-13 |  | 4,320 | 335,112 | 0.164613 | 0.0943661 | 0.0810868 |
| 2 | rs144843975 | T | C | 0.3683 | 0.06208 | 2.98E-09 |  | 4,320 | 335,112 | -0.222661 | 0.28719 | 0.438158 |
| 3 | rs4431216 | C | T | -0.151 | 0.02872 | 1.46E-07 |  | 4,320 | 335,112 | -0.0773065 | 0.0265337 | 0.0035738 |
| 4 | rs59715840 | C | T | 0.3368 | 0.06576 | 3.03E-07 |  | 4,320 | 335,112 | 0.0143293 | 0.0423422 | 0.73505 |
| 5 | rs62045817 | T | C | -0.2143 | 0.0366 | 4.76E-09 |  | 4,320 | 335,112 | -0.0359912 | 0.0340011 | 0.289813 |
| 6 | rs722926 | T | C | 0.1525 | 0.02762 | 3.36E-08 |  | 4,320 | 335,112 | 0.00237966 | 0.0238026 | 0.920364 |

SNP, single nucleotide polymorphism; EA, effect allele; OA, other allele; SE, standard error; T1D, type 1 diabetes.

**Supplementary Table S1.6** Information of identified SNPs in exposure (SSC-A on plasmacytoid DC) and outcomes (T1D).

|  |  | | | **Exposure (SSC-A on plasmacytoid DC)** | | |  | **Outcome (T1D)** | | | | |
| --- | --- | --- | --- | --- | --- | --- | --- | --- | --- | --- | --- | --- |
|  | **SNP** | **EA** | **OA** | **β** | **SE** | ***p* value** |  | **Case** | **Control** | **β** | **SE** | ***p* value** |
| 1 | rs117067704 | G | A | 0.4562 | 0.08992 | 3.91E-07 |  | 4,320 | 335,112 | 0.164613 | 0.0943661 | 0.0810868 |
| 2 | rs142456232 | T | C | -0.3341 | 0.05919 | 1.66E-08 |  | 4,320 | 335,112 | -0.192591 | 0.112266 | 0.0862561 |
| 3 | rs2903918 | G | C | -0.2195 | 0.02892 | 3.20E-14 |  | 4,320 | 335,112 | -0.0718631 | 0.0264135 | 0.00651433 |
| 4 | rs55733613 | C | G | -0.1957 | 0.03819 | 2.99E-07 |  | 4,320 | 335,112 | -0.0104954 | 0.0475701 | 0.82538 |
| 5 | rs6819052 | C | T | 0.1477 | 0.02811 | 1.49E-07 |  | 4,320 | 335,112 | -0.00669604 | 0.0248121 | 0.78726 |
| 6 | rs722926 | T | C | 0.1642 | 0.02772 | 3.15E-09 |  | 4,320 | 335,112 | 0.00237966 | 0.0238026 | 0.920364 |
| 7 | rs78736654 | G | T | -0.4036 | 0.07353 | 4.04E-08 |  | 4,320 | 335,112 | 0.0464462 | 0.10738 | 0.665347 |

SNP, single nucleotide polymorphism; EA, effect allele; OA, other allele; SE, standard error; T1D, type 1 diabetes.

**Supplementary Table S1.7** Information of identified SNPs in exposure (IgD+ CD38dim %lymphocyte) and outcomes (T1D).

|  |  | | | **Exposure (IgD+ CD38dim %lymphocyte)** | | |  | **Outcome (T1D)** | | | | |
| --- | --- | --- | --- | --- | --- | --- | --- | --- | --- | --- | --- | --- |
|  | **SNP** | **EA** | **OA** | **β** | **SE** | ***p* value** |  | **Case** | **Control** | **β** | **SE** | ***p* value** |
| 1 | rs12874404 | G | A | 0.1366 | 0.02617 | 1.79E-07 |  | 4,320 | 335,112 | 0.0660097 | 0.0625782 | 0.2915 |
| 2 | rs143109147 | C | T | 2.959 | 0.5843 | 4.10E-07 |  | 4,320 | 335,112 | 0.125138 | 0.0639092 | 0.0502227 |
| 3 | rs655580 | A | G | 0.1258 | 0.02423 | 2.08E-07 |  | 4,320 | 335,112 | -0.00707263 | 0.0237808 | 0.766154 |
| 4 | rs7840067 | C | T | -0.1537 | 0.03026 | 3.79E-07 |  | 4,320 | 335,112 | -0.026491 | 0.0271471 | 0.329147 |

SNP, single nucleotide polymorphism; EA, effect allele; OA, other allele; SE, standard error; T1D, type 1 diabetes.

**Supplementary Table S2** The results of MR-Egger intercept analysis.

| **Exposure** | **Outcome** | **Egger_intercept** | **SE** | ***p* value** |
| --- | --- | --- | --- | --- |
| CD28 on CD28+ CD45RA+ CD8br | T1D | -0.073108726 | 0.038272594 | 0.1962904 |
| CD80 on monocyte | T1D | -0.000321245 | 0.034985728 | 0.992930006 |
| EM CD8br %T cell | T1D | 0.009037838 | 0.025616533 | 0.747561973 |
| EM DN (CD4-CD8-) %T cell | T1D | -0.017787699 | 0.037755841 | 0.669693501 |
| FSC-A on plasmacytoid DC | T1D | 0.015412885 | 0.04517964 | 0.750159789 |
| SSC-A on plasmacytoid DC | T1D | -0.075150758 | 0.0472645 | 0.172704657 |
| IgD+ CD38dim %lymphocyte | T1D | 0.006267064 | 0.018306946 | 0.76472913 |

T1D, type 1 diabetes.

**Supplementary Table S3** The results of Cochran's Q analysis.

| **Exposure** | **Outcome** | **Method** | **Q** | **Q_df** | **Q_*p* val** |
| --- | --- | --- | --- | --- | --- |
| CD28 on CD28+ CD45RA+ CD8br | T1D | MR Egger | 2.885037201 | 2 | 0.236331783 |
| CD28 on CD28+ CD45RA+ CD8br | T1D | Inverse variance weighted | 8.148651413 | 3 | 0.043037381 |
| CD80 on monocyte | T1D | MR Egger | 2.519154693 | 7 | 0.925647804 |
| CD80 on monocyte | T1D | Inverse variance weighted | 2.519239005 | 8 | 0.960827758 |
| EM CD8br %T cell | T1D | MR Egger | 0.865515505 | 3 | 0.83374071 |
| EM CD8br %T cell | T1D | Inverse variance weighted | 0.989992293 | 4 | 0.911309669 |
| EM DN (CD4-CD8-) %T cell | T1D | MR Egger | 3.158039112 | 3 | 0.367894619 |
| EM DN (CD4-CD8-) %T cell | T1D | Inverse variance weighted | 3.391690032 | 4 | 0.494536998 |
| FSC-A on plasmacytoid DC | T1D | MR Egger | 6.92389609 | 4 | 0.139965041 |
| FSC-A on plasmacytoid DC | T1D | Inverse variance weighted | 7.125348687 | 5 | 0.211483648 |
| SSC-A on plasmacytoid DC | T1D | MR Egger | 5.430149097 | 5 | 0.365666092 |
| SSC-A on plasmacytoid DC | T1D | Inverse variance weighted | 8.175755914 | 6 | 0.225507749 |
| IgD+ CD38dim %lymphocyte | T1D | MR Egger | 1.617510812 | 2 | 0.445412079 |
| IgD+ CD38dim %lymphocyte | T1D | Inverse variance weighted | 1.73470241 | 3 | 0.629246414 |

T1D, type 1 diabetes.
